# Supplementary material for: Genetic Mapping of Seven Kinds of Locus for Resistance to Asian Soybean Rust
Source: Plants (Basel). 2023 Jun 9;12(12):2263. doi: 10.3390/plants12122263 (PMC10303739; doi:10.3390/plants12122263)
Supplement: Supplementary file 1 [file plants-12-02263-s001.zip › Figure_S1.pptx]

## Slide 1
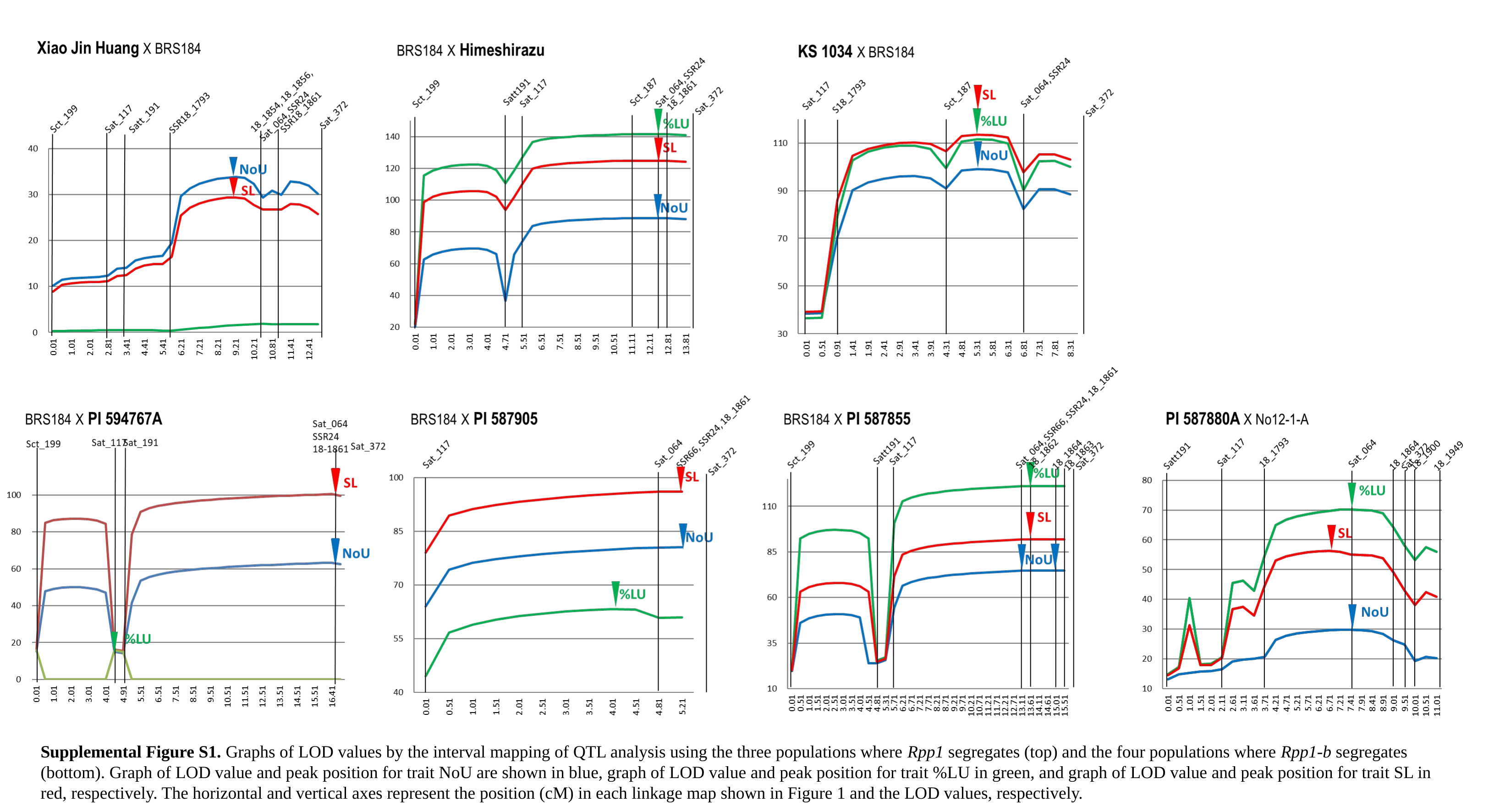

Supplemental Figure S1. Graphs of LOD values by the interval mapping of QTL analysis using the three populations where Rpp1 segregates (top) and the four populations where Rpp1-b segregates (bottom). Graph of LOD value and peak position for trait NoU are shown in blue, graph of LOD value and peak position for trait %LU in green, and graph of LOD value and peak position for trait SL in red, respectively. The horizontal and vertical axes represent the position (cM) in each linkage map shown in Figure 1 and the LOD values, respectively.
